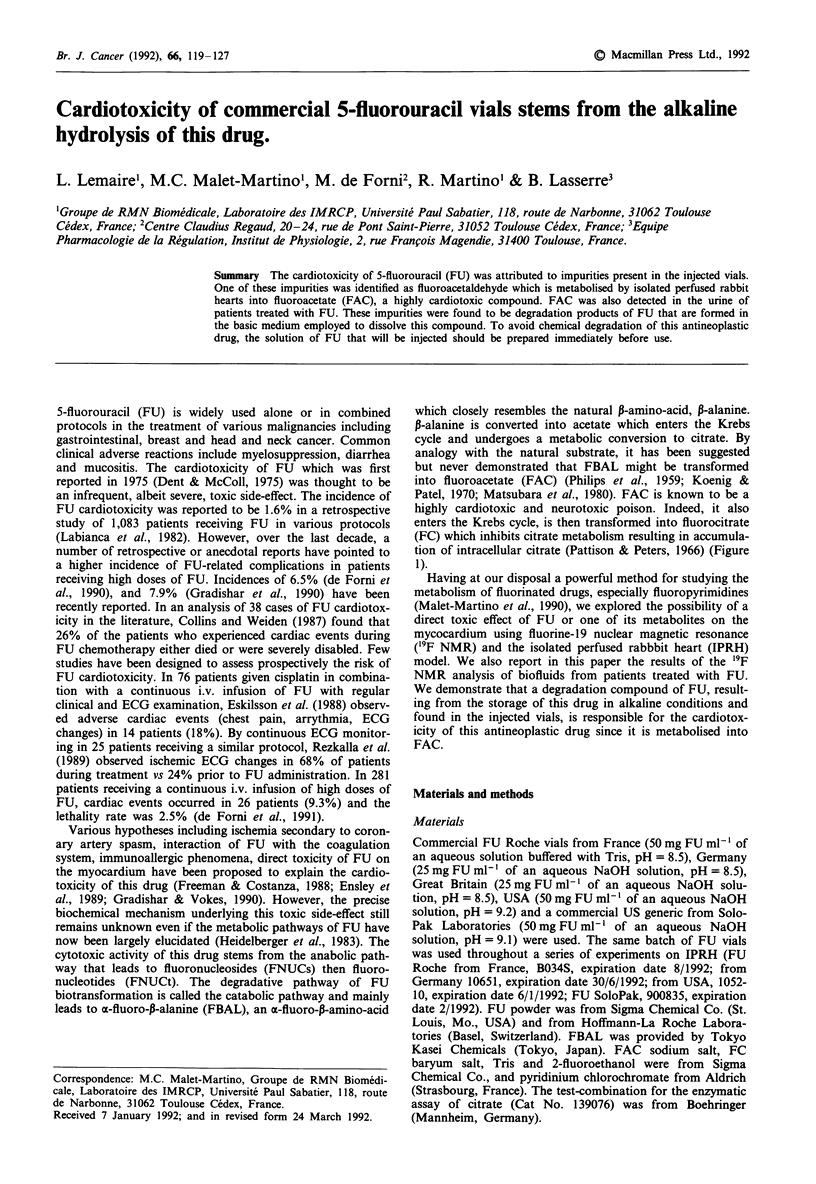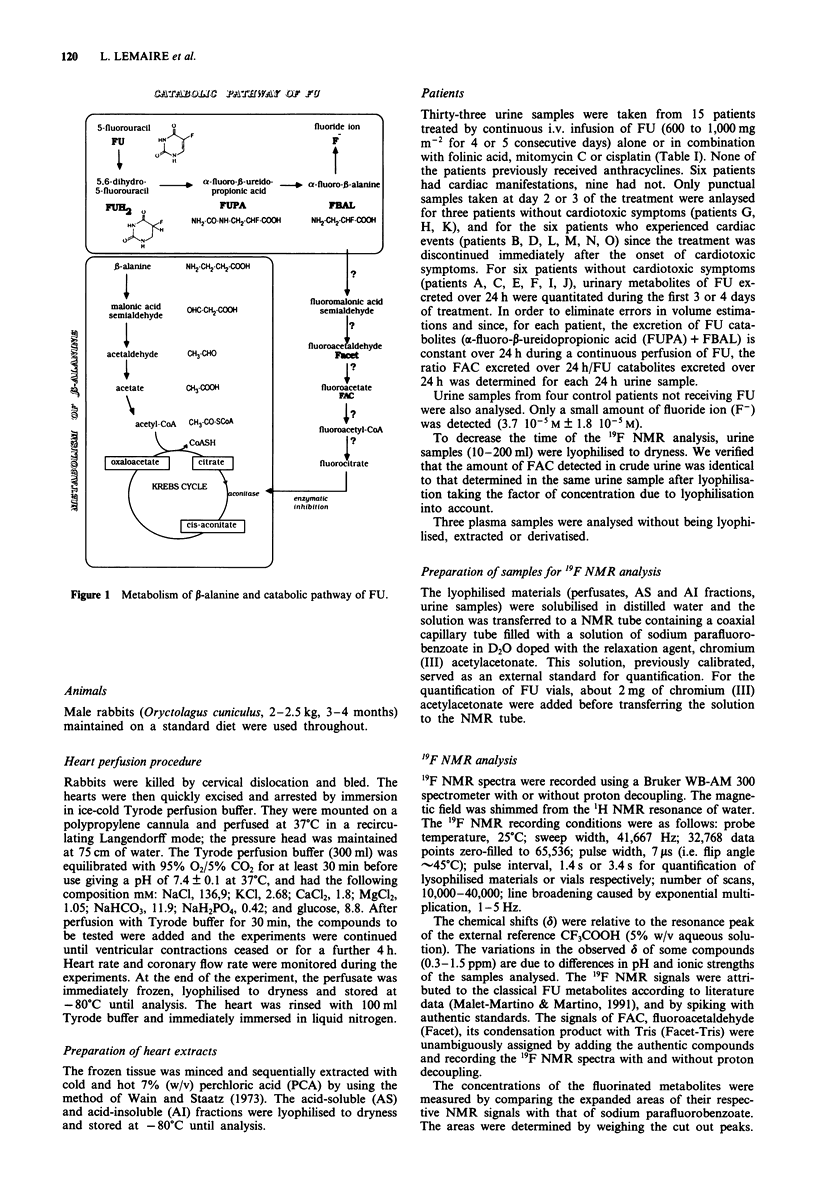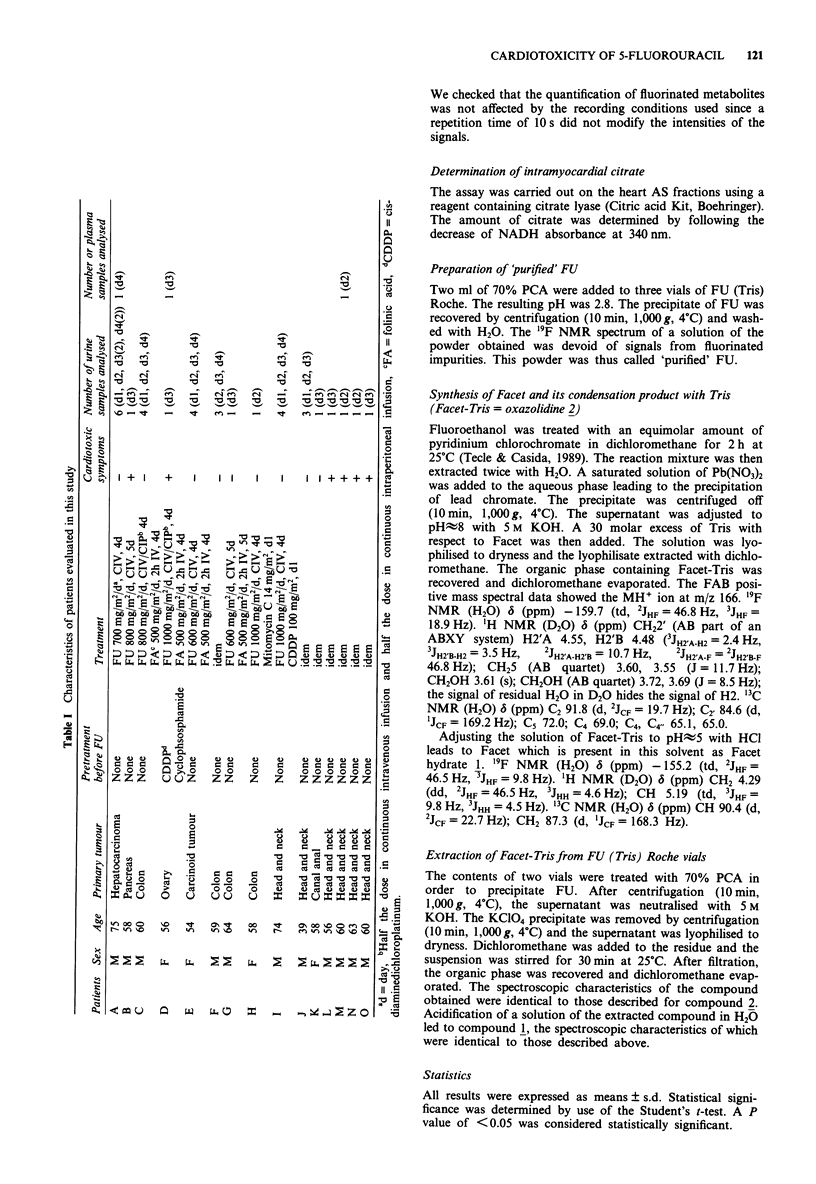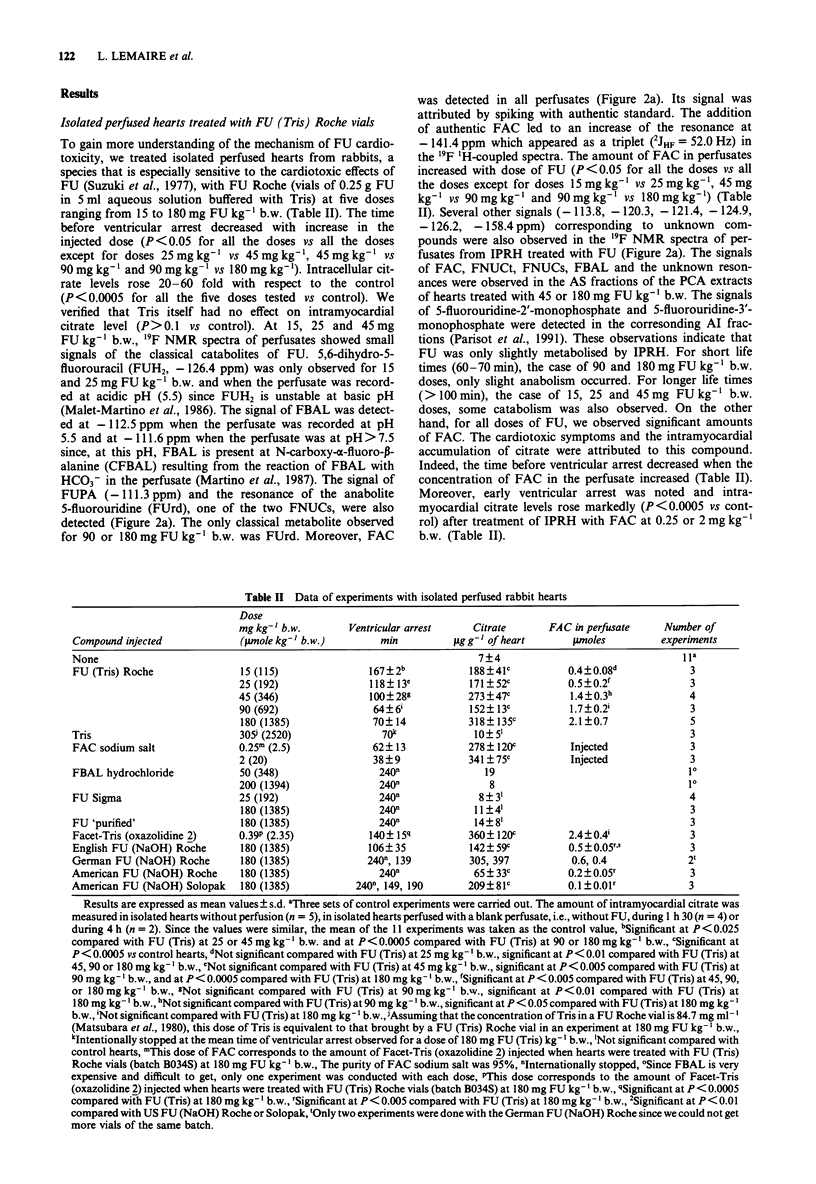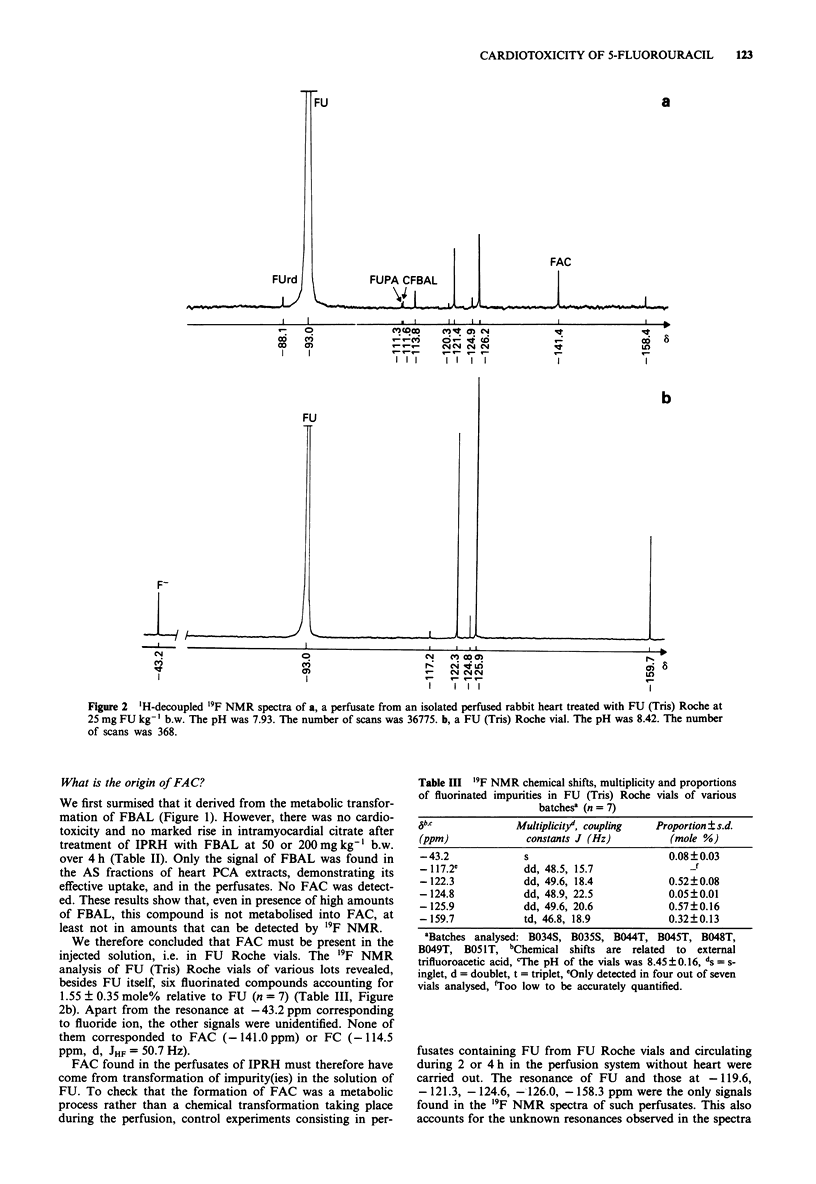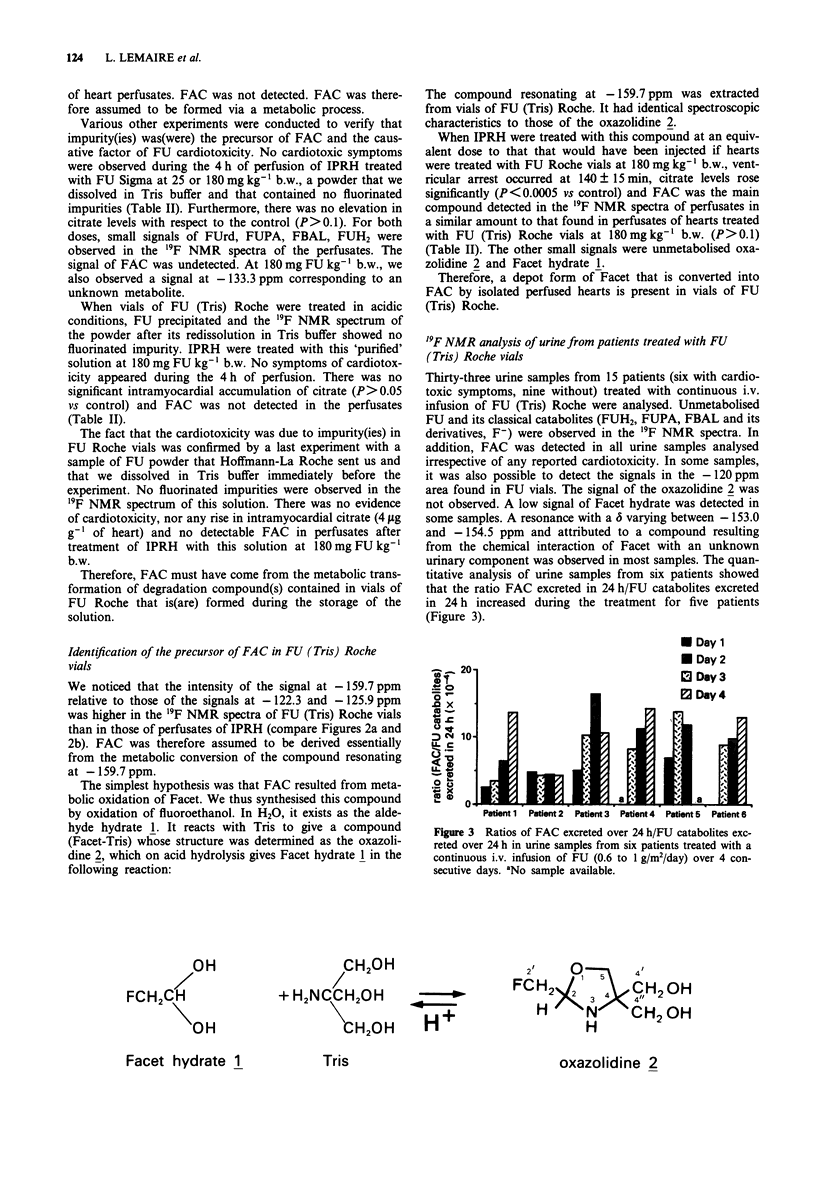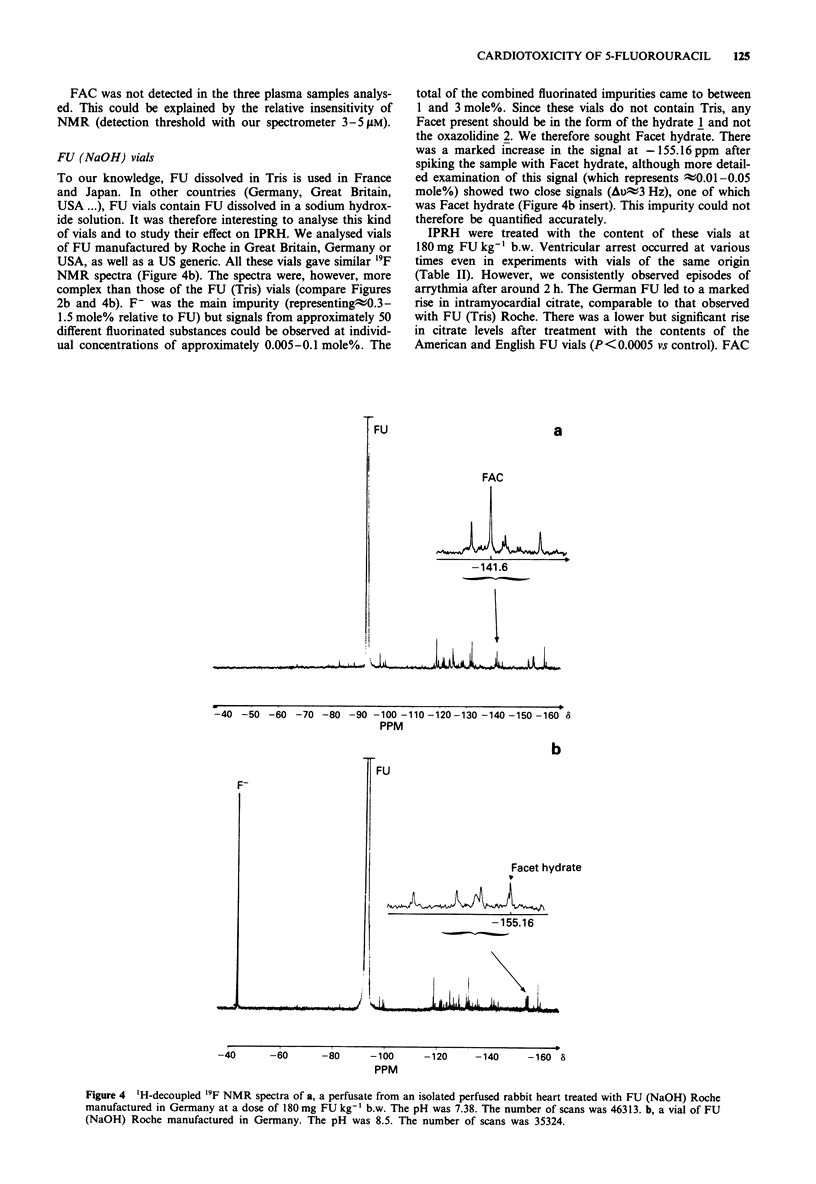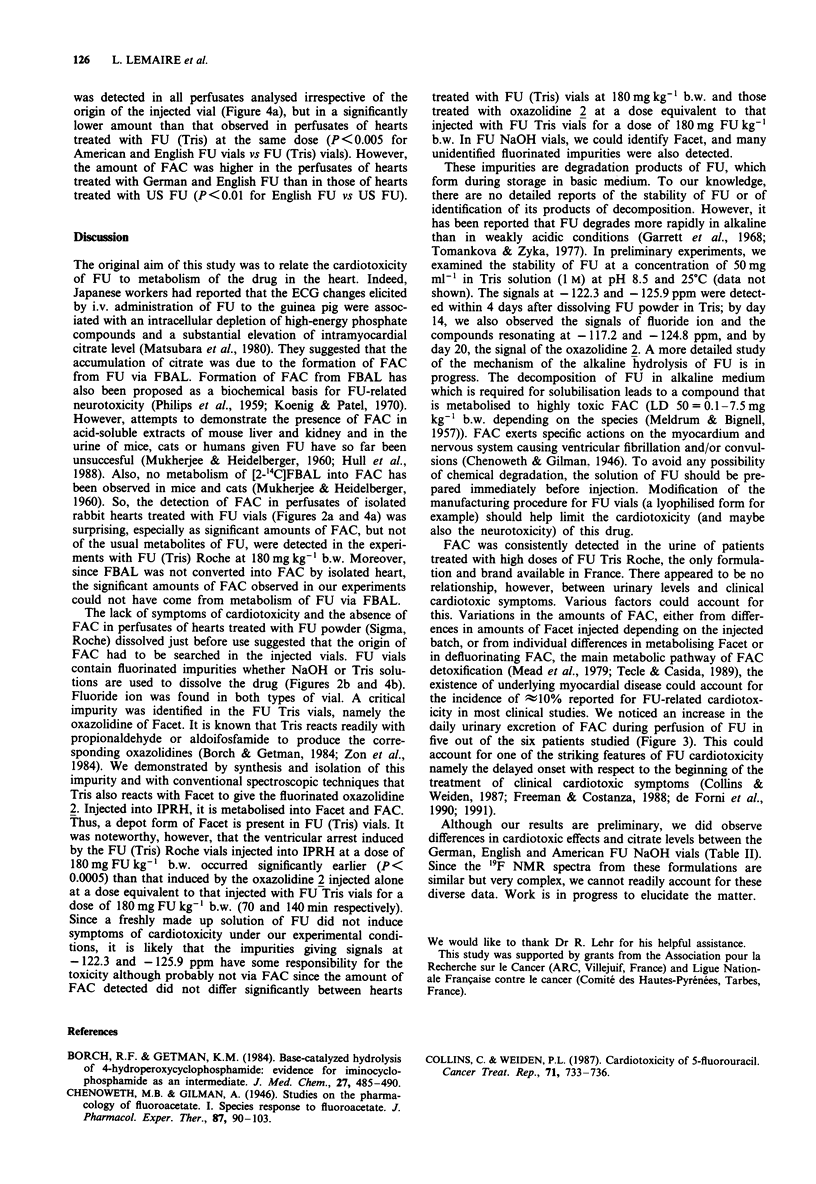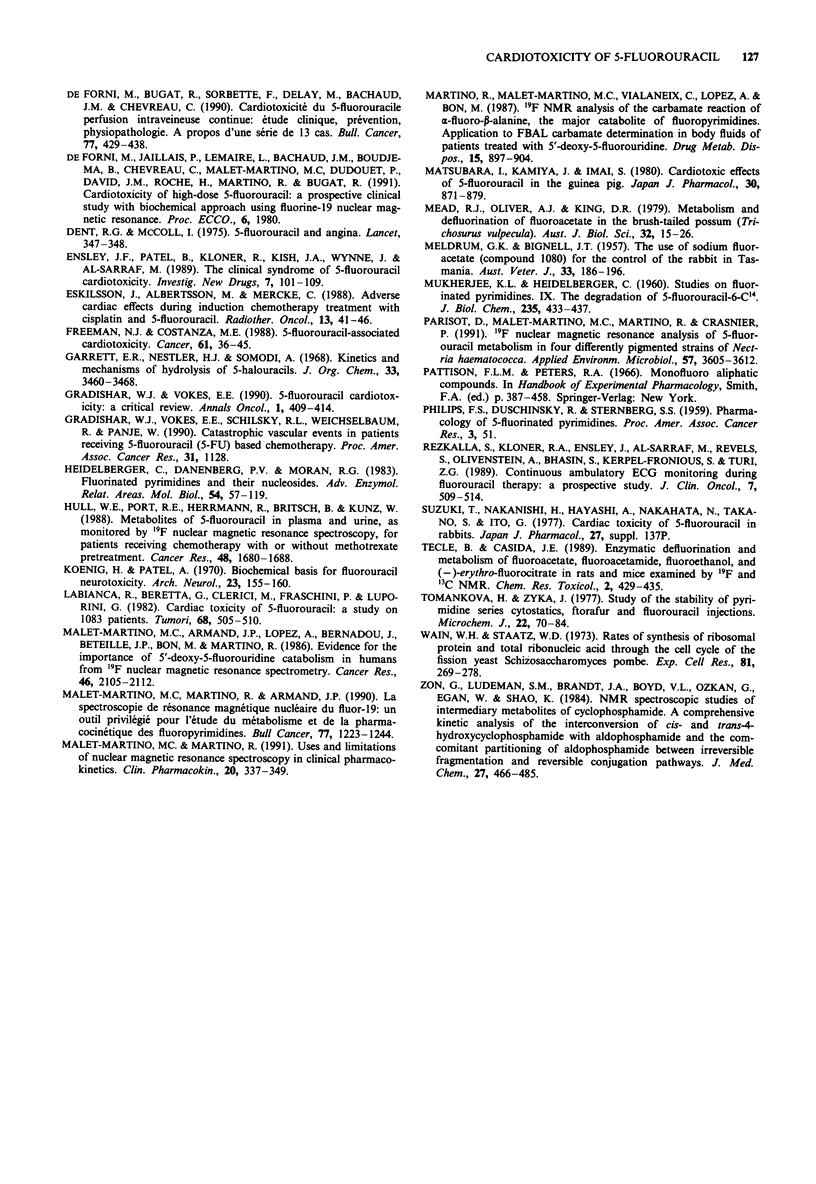# Cardiotoxicity of commercial 5-fluorouracil vials stems from the alkaline hydrolysis of this drug.

**DOI:** 10.1038/bjc.1992.227

**Published:** 1992-07

**Authors:** L. Lemaire, M. C. Malet-Martino, M. de Forni, R. Martino, B. Lasserre

**Affiliations:** Laboratoire des IMRCP, Université Paul Sabatier, Toulouse, France.

## Abstract

The cardiotoxicity of 5-fluorouracil (FU) was attributed to impurities present in the injected vials. One of these impurities was identified as fluoroacetaldehyde which is metabolised by isolated perfused rabbit hearts into fluoroacetate (FAC), a highly cardiotoxic compound. FAC was also detected in the urine of patients treated with FU. These impurities were found to be degradation products of FU that are formed in the basic medium employed to dissolve this compound. To avoid chemical degradation of this antineoplastic drug, the solution of FU that will be injected should be prepared immediately before use.


 © Macmillan Press Ltd., 1992

### Cardiotoxicity of commercial 5-fluorouracil vials stems from the alkaline hydrolysis of this drug.

L. Lemaire[1], M.C. Malet-Martino[1], M. de Forni[2], R. Martino[1] & B. Lasserre[3]

[1]*Groupe de RMN Biomédicale, Laboratoire des IMRCP, Université Paul Sabatier, 118, route de Narbonne, 31062 Toulouse Cédex, France;* [2]*Centre Claudius Regaud, 20-24, rue de Pont Saint-Pierre, 31052 Toulouse Cédex, France;* [3]*Equipe Pharmacologie de la Régulation, Institut de Physiologie, 2, rue François Magendie, 31400 Toulouse, France.*

**Summary** The cardiotoxicity of 5-fluorouracil (FU) was attributed to impurities present in the injected vials. One of these impurities was identified as fluoroacetaldehyde which is metabolised by isolated perfused rabbit hearts into fluoroacetate (FAC), a highly cardiotoxic compound. FAC was also detected in the urine of patients treated with FU. These impurities were found to be degradation products of FU that are formed in the basic medium employed to dissolve this compound. To avoid chemical degradation of this antineoplastic drug, the solution of FU that will be injected should be prepared immediately before use.

5-fluorouracil (FU) is widely used alone or in combined protocols in the treatment of various malignancies including gastrointestinal, breast and head and neck cancer. Common clinical adverse reactions include myelosuppression, diarrhea and mucositis. The cardiotoxicity of FU which was first reported in 1975 (Dent & McColl, 1975) was thought to be an infrequent, albeit severe, toxic side-effect. The incidence of FU cardiotoxicity was reported to be 1.6% in a retrospective study of 1,083 patients receiving FU in various protocols (Labianca *et al.*, 1982). However, over the last decade, a number of retrospective or anecdotal reports have pointed to a higher incidence of FU-related complications in patients receiving high doses of FU. Incidences of 6.5% (de Forni *et al.*, 1990), and 7.9% (Gradishar *et al.*, 1990) have been recently reported. In an analysis of 38 cases of FU cardiotoxicity in the literature, Collins and Weiden (1987) found that 26% of the patients who experienced cardiac events during FU chemotherapy either died or were severely disabled. Few studies have been designed to assess prospectively the risk of FU cardiotoxicity. In 76 patients given cisplatin in combination with a continuous i.v. infusion of FU with regular clinical and ECG examination, Eskilsson *et al.* (1988) observed adverse cardiac events (chest pain, arrythmia, ECG changes) in 14 patients (18%). By continuous ECG monitoring in 25 patients receiving a similar protocol, Rezkalla *et al.* (1989) observed ischemic ECG changes in 68% of patients during treatment *vs* 24% prior to FU administration. In 281 patients receiving a continuous i.v. infusion of high doses of FU, cardiac events occurred in 26 patients (9.3%) and the lethality rate was 2.5% (de Forni *et al.*, 1991).

Various hypotheses including ischemia secondary to coronary artery spasm, interaction of FU with the coagulation system, immunoallergic phenomena, direct toxicity of FU on the myocardium have been proposed to explain the cardiotoxicity of this drug (Freeman & Costanza, 1988; Ensley *et al.*, 1989; Gradishar & Vokes, 1990). However, the precise biochemical mechanism underlying this toxic side-effect still remains unknown even if the metabolic pathways of FU have now been largely elucidated (Heidelberger *et al.*, 1983). The cytotoxic activity of this drug stems from the anabolic pathway that leads to fluoronucleosides (FNUCs) then fluoronucleotides (FNUCt). The degradative pathway of FU biotransformation is called the catabolic pathway and mainly leads to α-fluoro-β-alanine (FBAL), an α-fluoro-β-amino-acid which closely resembles the natural β-amino-acid, β-alanine. β-alanine is converted into acetate which enters the Krebs cycle and undergoes a metabolic conversion to citrate. By analogy with the natural substrate, it has been suggested but never demonstrated that FBAL might be transformed into fluoroacetate (FAC) (Philips *et al.*, 1959; Koenig & Patel, 1970; Matsubara *et al.*, 1980). FAC is known to be a highly cardiotoxic and neurotoxic poison. Indeed, it also enters the Krebs cycle, is then transformed into fluorocitrate (FC) which inhibits citrate metabolism resulting in accumulation of intracellular citrate (Pattison & Peters, 1966) (Figure 1).

Having at our disposal a powerful method for studying the metabolism of fluorinated drugs, especially fluoropyrimidines (Malet-Martino *et al.*, 1990), we explored the possibility of a direct toxic effect of FU or one of its metabolites on the mycocardium using fluorine-19 nuclear magnetic resonance ($^{19}F$ NMR) and the isolated perfused rabbbit heart (IPRH) model. We also report in this paper the results of the $^{19}F$ NMR analysis of biofluids from patients treated with FU. We demonstrate that a degradation compound of FU, resulting from the storage of this drug in alkaline conditions and found in the injected vials, is responsible for the cardiotoxicity of this antineoplastic drug since it is metabolised into FAC.

Correspondence: M.C. Malet-Martino, Groupe de RMN Biomédicale, Laboratoire des IMRCP, Université Paul Sabatier, 118, route de Narbonne, 31062 Toulouse Cédex, France.
Received 7 January 1992; and in revised form 24 March 1992.

### Materials and methods

### *Materials*

Commercial FU Roche vials from France (50 mg FU $ml^{-1}$ of an aqueous solution buffered with Tris, pH = 8.5), Germany (25 mg FU $ml^{-1}$ of an aqueous NaOH solution, pH = 8.5), Great Britain (25 mg FU $ml^{-1}$ of an aqueous NaOH solution, pH = 8.5), USA (50 mg FU $ml^{-1}$ of an aqueous NaOH solution, pH = 9.2) and a commercial US generic from SoloPak Laboratories (50 mg FU $ml^{-1}$ of an aqueous NaOH solution, pH = 9.1) were used. The same batch of FU vials was used throughout a series of experiments on IPRH (FU Roche from France, B034S, expiration date 8/1992; from Germany 10651, expiration date 30/6/1992; from USA, 1052-10, expiration date 6/1/1992; FU SoloPak, 900835, expiration date 2/1992). FU powder was from Sigma Chemical Co. (St. Louis, Mo., USA) and from Hoffmann-La Roche Laboratories (Basel, Switzerland). FBAL was provided by Tokyo Kasei Chemicals (Tokyo, Japan). FAC sodium salt, FC baryum salt, Tris and 2-fluoroethanol were from Sigma Chemical Co., and pyridinium chlorochromate from Aldrich (Strasbourg, France). The test-combination for the enzymatic assay of citrate (Cat No. 139076) was from Boehringer (Mannheim, Germany).

**Figure 1** Metabolism of β-alanine and catabolic pathway of FU.

*Animals*

Male rabbits (*Oryctolagus cuniculus*, 2–2.5 kg, 3–4 months) maintained on a standard diet were used throughout.

*Heart perfusion procedure*

Rabbits were killed by cervical dislocation and bled. The hearts were then quickly excised and arrested by immersion in ice-cold Tyrode perfusion buffer. They were mounted on a polypropylene cannula and perfused at 37°C in a recirculating Langendorff mode; the pressure head was maintained at 75 cm of water. The Tyrode perfusion buffer (300 ml) was equilibrated with 95% $O_2$/5% $CO_2$ for at least 30 min before use giving a pH of 7.4 ± 0.1 at 37°C, and had the following composition mM: NaCl, 136,9; KCl, 2.68; $CaCl_2$, 1.8; $MgCl_2$, 1.05; $NaHCO_3$, 11.9; $NaH_2PO_4$, 0.42; and glucose, 8.8. After perfusion with Tyrode buffer for 30 min, the compounds to be tested were added and the experiments were continued until ventricular contractions ceased or for a further 4 h. Heart rate and coronary flow rate were monitored during the experiments. At the end of the experiment, the perfusate was immediately frozen, lyophilised to dryness and stored at −80°C until analysis. The heart was rinsed with 100 ml Tyrode buffer and immediately immersed in liquid nitrogen.

*Preparation of heart extracts*

The frozen tissue was minced and sequentially extracted with cold and hot 7% (w/v) perchloric acid (PCA) by using the method of Wain and Staatz (1973). The acid-soluble (AS) and acid-insoluble (AI) fractions were lyophilised to dryness and stored at −80°C until analysis.

*Patients*

Thirty-three urine samples were taken from 15 patients treated by continuous i.v. infusion of FU (600 to 1,000 mg $m^{-2}$ for 4 or 5 consecutive days) alone or in combination with folinic acid, mitomycin C or cisplatin (Table I). None of the patients previously received anthracyclines. Six patients had cardiac manifestations, nine had not. Only punctual samples taken at day 2 or 3 of the treatment were anlaysed for three patients without cardiotoxic symptoms (patients G, H, K), and for the six patients who experienced cardiac events (patients B, D, L, M, N, O) since the treatment was discontinued immediately after the onset of cardiotoxic symptoms. For six patients without cardiotoxic symptoms (patients A, C, E, F, I, J), urinary metabolites of FU excreted over 24 h were quantitated during the first 3 or 4 days of treatment. In order to eliminate errors in volume estimations and since, for each patient, the excretion of FU catabolites (α-fluoro-β-ureidopropionic acid (FUPA) + FBAL) is constant over 24 h during a continuous perfusion of FU, the ratio FAC excreted over 24 h/FU catabolites excreted over 24 h was determined for each 24 h urine sample.

Urine samples from four control patients not receiving FU were also analysed. Only a small amount of fluoride ion ($F^-$) was detected (3.7 $10^{-5}$ M ± 1.8 $10^{-5}$ M).

To decrease the time of the $^{19}F$ NMR analysis, urine samples (10–200 ml) were lyophilised to dryness. We verified that the amount of FAC detected in crude urine was identical to that determined in the same urine sample after lyophilisation taking the factor of concentration due to lyophilisation into account.

Three plasma samples were analysed without being lyophilised, extracted or derivatised.

*Preparation of samples for $^{19}F$ NMR analysis*

The lyophilised materials (perfusates, AS and AI fractions, urine samples) were solubilised in distilled water and the solution was transferred to a NMR tube containing a coaxial capillary tube filled with a solution of sodium parafluorobenzoate in $D_2O$ doped with the relaxation agent, chromium (III) acetylacetonate. This solution, previously calibrated, served as an external standard for quantification. For the quantification of FU vials, about 2 mg of chromium (III) acetylacetonate were added before transferring the solution to the NMR tube.

*$^{19}F$ NMR analysis*

$^{19}F$ NMR spectra were recorded using a Bruker WB-AM 300 spectrometer with or without proton decoupling. The magnetic field was shimmed from the $^1H$ NMR resonance of water. The $^{19}F$ NMR recording conditions were as follows: probe temperature, 25°C; sweep width, 41,667 Hz; 32,768 data points zero-filled to 65,536; pulse width, 7 μs (i.e. flip angle ~45°C); pulse interval, 1.4 s or 3.4 s for quantification of lysophilised materials or vials respectively; number of scans, 10,000–40,000; line broadening caused by exponential multiplication, 1–5 Hz.

The chemical shifts (δ) were relative to the resonance peak of the external reference $CF_3COOH$ (5% w/v aqueous solution). The variations in the observed δ of some compounds (0.3–1.5 ppm) are due to differences in pH and ionic strengths of the samples analysed. The $^{19}F$ NMR signals were attributed to the classical FU metabolites according to literature data (Malet-Martino & Martino, 1991), and by spiking with authentic standards. The signals of FAC, fluoroacetaldehyde (Facet), its condensation product with Tris (Facet-Tris) were unambiguously assigned by adding the authentic compounds and recording the $^{19}F$ NMR spectra with and without proton decoupling.

The concentrations of the fluorinated metabolites were measured by comparing the expanded areas of their respective NMR signals with that of sodium parafluorobenzoate. The areas were determined by weighing the cut out peaks.

**Table I** Characteristics of patients evaluated in this study

| Patients | Sex | Age | Primary tumour | Pretreatment before FU | Treatment | Cardiotoxic symptoms | Number of urine samples analysed | Number of plasma samples analysed |
|---|---|---|---|---|---|---|---|---|
| A | M | 75 | Hepatocarcinoma | None | FU 700 mg/m²/d[a], CIV, 4d | – | 6 (d1, d2, d3(2), d4(2)) | 1 (d4) |
| B | M | 58 | Pancreas | None | FU 800 mg/m²/d, CIV, 5d | + | 1 (d3) | |
| C | M | 60 | Colon | None | FU 800 mg/m²/d, CIV/CIP[b] 4d<br>FA[c] 500 mg/m²/d, 2h IV, 4d | – | 4 (d1, d2, d3, d4) | |
| D | F | 56 | Ovary | CDDP[d]<br>Cyclophsosphamide | FU 1000 mg/m²/d, CIV/CIP[b], 4d<br>FA 500 mg/m²/d, 2h IV, 4d | + | 1 (d3) | 1 (d3) |
| E | F | 54 | Carcinoid tumour | None | FU 600 mg/m²/d, CIV, 4d<br>FA 500 mg/m²/d, 2h IV, 4d | – | 4 (d1, d2, d3, d4) | |
| F | M | 59 | Colon | None | idem | – | 3 (d2, d3, d4) | |
| G | M | 64 | Colon | None | FU 600 mg/m²/d, CIV, 5d<br>FA 500 mg/m²/d, 2h IV, 5d | – | 1 (d3) | |
| H | F | 58 | Colon | None | FU 1000 mg/m²/d, CIV, 4d<br>Mitomycin C 14 mg/m², d1 | – | 1 (d2) | |
| I | M | 74 | Head and neck | None | FU 1000 mg/m²/d, CIV, 4d<br>CDDP 100 mg/m², d1 | – | 4 (d1, d2, d3, d4) | |
| J | M | 39 | Head and neck | None | idem | – | 3 (d1, d2, d3) | |
| K | F | 58 | Canal anal | None | idem | – | 1 (d3) | |
| L | M | 56 | Head and neck | None | idem | + | 1 (d3) | |
| M | M | 60 | Head and neck | None | idem | + | 1 (d2) | 1 (d2) |
| N | M | 63 | Head and neck | None | idem | + | 1 (d2) | |
| O | M | 60 | Head and neck | None | idem | + | 1 (d3) | |

[a]d = day, [b]Half the dose in continuous intravenous infusion and half the dose in continuous intraperitoneal infusion, [c]FA = folinic acid, [d]CDDP = cis-diaminedichloroplatinum.

We checked that the quantification of fluorinated metabolites was not affected by the recording conditions used since a repetition time of 10 s did not modify the intensities of the signals.

### *Determination of intramyocardial citrate*

The assay was carried out on the heart AS fractions using a reagent containing citrate lyase (Citric acid Kit, Boehringer). The amount of citrate was determined by following the decrease of NADH absorbance at 340 nm.

### *Preparation of 'purified' FU*

Two ml of 70% PCA were added to three vials of FU (Tris) Roche. The resulting pH was 2.8. The precipitate of FU was recovered by centrifugation (10 min, 1,000 *g*, 4°C) and washed with $H_2O$. The $^{19}F$ NMR spectrum of a solution of the powder obtained was devoid of signals from fluorinated impurities. This powder was thus called 'purified' FU.

### *Synthesis of Facet and its condensation product with Tris (Facet-Tris = oxazolidine 2)*

Fluoroethanol was treated with an equimolar amount of pyridinium chlorochromate in dichloromethane for 2 h at 25°C (Tecle & Casida, 1989). The reaction mixture was then extracted twice with $H_2O$. A saturated solution of $Pb(NO_3)_2$ was added to the aqueous phase leading to the precipitation of lead chromate. The precipitate was centrifuged off (10 min, 1,000 *g*, 4°C). The supernatant was adjusted to pH≈8 with 5 M KOH. A 30 molar excess of Tris with respect to Facet was then added. The solution was lyophilised to dryness and the lyophilisate extracted with dichloromethane. The organic phase containing Facet-Tris was recovered and dichloromethane evaporated. The FAB positive mass spectral data showed the $MH^+$ ion at m/z 166. $^{19}F$ NMR ($H_2O$) $\delta$ (ppm) −159.7 (td, $^2J_{HF} = 46.8$ Hz, $^3J_{HF} =$ 18.9 Hz). $^1H$ NMR ($D_2O$) $\delta$ (ppm) $CH_2 2'$ (AB part of an ABXY system) H2'A 4.55, H2'B 4.48 ($^3J_{H2'A\text{-}H2} = 2.4$ Hz, $^3J_{H2'B\text{-}H2} = 3.5$ Hz, $^2J_{H2'A\text{-}H2'B} = 10.7$ Hz, $^2J_{H2'A\text{-}F} = {}^2J_{H2'B\text{-}F}$ 46.8 Hz); $CH_2 5$ (AB quartet) 3.60, 3.55 (J = 11.7 Hz); $CH_2OH$ 3.61 (s); $CH_2OH$ (AB quartet) 3.72, 3.69 (J = 8.5 Hz); the signal of residual $H_2O$ in $D_2O$ hides the signal of H2. $^{13}C$ NMR ($H_2O$) $\delta$ (ppm) $C_2$ 91.8 (d, $^2J_{CF} = 19.7$ Hz); $C_{2'}$ 84.6 (d, $^1J_{CF} = 169.2$ Hz); $C_5$ 72.0; $C_4$ 69.0; $C_{4'}$, $C_{4''}$ 65.1, 65.0.

Adjusting the solution of Facet-Tris to pH≈5 with HCl leads to Facet which is present in this solvent as Facet hydrate 1. $^{19}F$ NMR ($H_2O$) $\delta$ (ppm) −155.2 (td, $^2J_{HF} =$ 46.5 Hz, $^3J_{HF} = 9.8$ Hz). $^1H$ NMR ($D_2O$) $\delta$ (ppm) $CH_2$ 4.29 (dd, $^2J_{HF} = 46.5$ Hz, $^3J_{HH} = 4.6$ Hz); CH 5.19 (td, $^3J_{HF} =$ 9.8 Hz, $^3J_{HH} = 4.5$ Hz). $^{13}C$ NMR ($H_2O$) $\delta$ (ppm) CH 90.4 (d, $^2J_{CF} = 22.7$ Hz); $CH_2$ 87.3 (d, $^1J_{CF} = 168.3$ Hz).

### *Extraction of Facet-Tris from FU (Tris) Roche vials*

The contents of two vials were treated with 70% PCA in order to precipitate FU. After centrifugation (10 min, 1,000 *g*, 4°C), the supernatant was neutralised with 5 M KOH. The $KClO_4$ precipitate was removed by centrifugation (10 min, 1,000 *g*, 4°C) and the supernatant was lyophilised to dryness. Dichloromethane was added to the residue and the suspension was stirred for 30 min at 25°C. After filtration, the organic phase was recovered and dichloromethane evaporated. The spectroscopic characteristics of the compound obtained were identical to those described for compound 2. Acidification of a solution of the extracted compound in $H_2O$ led to compound 1, the spectroscopic characteristics of which were identical to those described above.

### *Statistics*

All results were expressed as means ± s.d. Statistical significance was determined by use of the Student's *t*-test. A *P* value of $<0.05$ was considered statistically significant.

### Results

### *Isolated perfused hearts treated with FU (Tris) Roche vials*

To gain more understanding of the mechanism of FU cardiotoxicity, we treated isolated perfused hearts from rabbits, a species that is especially sensitive to the cardiotoxic effects of FU (Suzuki *et al.*, 1977), with FU Roche (vials of 0.25 g FU in 5 ml aqueous solution buffered with Tris) at five doses ranging from 15 to 180 mg FU $kg^{-1}$ b.w. (Table II). The time before ventricular arrest decreased with increase in the injected dose ($P<0.05$ for all the doses *vs* all the doses except for doses 25 mg $kg^{-1}$ *vs* 45 mg $kg^{-1}$, 45 mg $kg^{-1}$ *vs* 90 mg $kg^{-1}$ and 90 mg $kg^{-1}$ *vs* 180 mg $kg^{-1}$). Intracellular citrate levels rose 20–60 fold with respect to the control ($P<0.0005$ for all the five doses tested *vs* control). We verified that Tris itself had no effect on intramyocardial citrate level ($P>0.1$ *vs* control). At 15, 25 and 45 mg FU $kg^{-1}$ b.w., $^{19}F$ NMR spectra of perfusates showed small signals of the classical catabolites of FU. 5,6-dihydro-5-fluorouracil ($FUH_2$, −126.4 ppm) was only observed for 15 and 25 mg FU $kg^{-1}$ b.w. and when the perfusate was recorded at acidic pH (5.5) since $FUH_2$ is unstable at basic pH (Malet-Martino *et al.*, 1986). The signal of FBAL was detected at −112.5 ppm when the perfusate was recorded at pH 5.5 and at −111.6 ppm when the perfusate was at pH > 7.5 since, at this pH, FBAL is present at N-carboxy-α-fluoro-β-alanine (CFBAL) resulting from the reaction of FBAL with $HCO_3^-$ in the perfusate (Martino *et al.*, 1987). The signal of FUPA (−111.3 ppm) and the resonance of the anabolite 5-fluorouridine (FUrd), one of the two FNUCs, were also detected (Figure 2a). The only classical metabolite observed for 90 or 180 mg FU $kg^{-1}$ b.w. was FUrd. Moreover, FAC was detected in all perfusates (Figure 2a). Its signal was attributed by spiking with authentic standard. The addition of authentic FAC led to an increase of the resonance at −141.4 ppm which appeared as a triplet ($^2J_{HF} = 52.0$ Hz) in the $^{19}F$ $^1H$-coupled spectra. The amount of FAC in perfusates increased with dose of FU ($P<0.05$ for all the doses *vs* all the doses except for doses 15 mg $kg^{-1}$ *vs* 25 mg $kg^{-1}$, 45 mg $kg^{-1}$ *vs* 90 mg $kg^{-1}$ and 90 mg $kg^{-1}$ *vs* 180 mg $kg^{-1}$) (Table II). Several other signals (−113.8, −120.3, −121.4, −124.9, −126.2, −158.4 ppm) corresponding to unknown compounds were also observed in the $^{19}F$ NMR spectra of perfusates from IPRH treated with FU (Figure 2a). The signals of FAC, FNUCt, FNUCs, FBAL and the unknown resonances were observed in the AS fractions of the PCA extracts of hearts treated with 45 or 180 mg FU $kg^{-1}$ b.w. The signals of 5-fluorouridine-2′-monophosphate and 5-fluorouridine-3′-monophosphate were detected in the corresonding AI fractions (Parisot *et al.*, 1991). These observations indicate that FU was only slightly metabolised by IPRH. For short life times (60–70 min), the case of 90 and 180 mg FU $kg^{-1}$ b.w. doses, only slight anabolism occurred. For longer life times (> 100 min), the case of 15, 25 and 45 mg FU $kg^{-1}$ b.w. doses, some catabolism was also observed. On the other hand, for all doses of FU, we observed significant amounts of FAC. The cardiotoxic symptoms and the intramyocardial accumulation of citrate were attributed to this compound. Indeed, the time before ventricular arrest decreased when the concentration of FAC in the perfusate increased (Table II). Moreover, early ventricular arrest was noted and intramyocardial citrate levels rose markedly ($P<0.0005$ *vs* control) after treatment of IPRH with FAC at 0.25 or 2 mg $kg^{-1}$ b.w. (Table II).

**Table II** Data of experiments with isolated perfused rabbit hearts

| *Compound injected* | *Dose mg $kg^{-1}$ b.w. (μmole $kg^{-1}$ b.w.)* | *Ventricular arrest min* | *Citrate μg $g^{-1}$ of heart* | *FAC in perfusate μmoles* | *Number of experiments* |
|---|---|---|---|---|---|
| None | | | 7 ± 4 | | 11[a] |
| FU (Tris) Roche | 15 (115) | 167 ± 2[b] | 188 ± 41[c] | 0.4 ± 0.08[d] | 3 |
| | 25 (192) | 118 ± 13[e] | 171 ± 52[c] | 0.5 ± 0.2[f] | 3 |
| | 45 (346) | 100 ± 28[g] | 273 ± 47[c] | 1.4 ± 0.3[h] | 4 |
| | 90 (692) | 64 ± 6[i] | 152 ± 13[c] | 1.7 ± 0.2[i] | 3 |
| | 180 (1385) | 70 ± 14 | 318 ± 135[c] | 2.1 ± 0.7 | 5 |
| Tris | 305[j] (2520) | 70[k] | 10 ± 5[l] | | 3 |
| FAC sodium salt | 0.25[m] (2.5) | 62 ± 13 | 278 ± 120[c] | Injected | 3 |
| | 2 (20) | 38 ± 9 | 341 ± 75[c] | Injected | 3 |
| FBAL hydrochloride | 50 (348) | 240[n] | 19 | | 1[o] |
| | 200 (1394) | 240[n] | 8 | | 1[o] |
| FU Sigma | 25 (192) | 240[n] | 8 ± 3[l] | | 4 |
| | 180 (1385) | 240[n] | 11 ± 4[l] | | 3 |
| FU 'purified' | 180 (1385) | 240[n] | 14 ± 8[l] | | 3 |
| Facet-Tris (oxazolidine 2) | 0.39[p] (2.35) | 140 ± 15[q] | 360 ± 120[c] | 2.4 ± 0.4[i] | 3 |
| English FU (NaOH) Roche | 180 (1385) | 106 ± 35 | 142 ± 59[c] | 0.5 ± 0.05[r,s] | 3 |
| German FU (NaOH) Roche | 180 (1385) | 240[n], 139 | 305, 397 | 0.6, 0.4 | 2[t] |
| American FU (NaOH) Roche | 180 (1385) | 240[n] | 65 ± 33[c] | 0.2 ± 0.05[r] | 3 |
| American FU (NaOH) Solopak | 180 (1385) | 240[n], 149, 190 | 209 ± 81[c] | 0.1 ± 0.01[r] | 3 |

Results are expressed as mean values ± s.d. [a]Three sets of control experiments were carried out. The amount of intramyocardial citrate was measured in isolated hearts without perfusion ($n = 5$), in isolated hearts perfused with a blank perfusate, i.e., without FU, during 1 h 30 ($n = 4$) or during 4 h ($n = 2$). Since the values were similar, the mean of the 11 experiments was taken as the control value, [b]Significant at $P<0.025$ compared with FU (Tris) at 25 or 45 mg $kg^{-1}$ b.w. and at $P<0.0005$ compared with FU (Tris) at 90 or 180 mg $kg^{-1}$ b.w., [c]Significant at $P<0.0005$ *vs* control hearts, [d]Not significant compared with FU (Tris) at 25 mg $kg^{-1}$ b.w., significant at $P<0.01$ compared with FU (Tris) at 45, 90 or 180 mg $kg^{-1}$ b.w., [e]Not significant compared with FU (Tris) at 45 mg $kg^{-1}$ b.w., significant at $P<0.005$ compared with FU (Tris) at 90 mg $kg^{-1}$ b.w., and at $P<0.0005$ compared with FU (Tris) at 180 mg $kg^{-1}$ b.w., [f]Significant at $P<0.005$ compared with FU (Tris) at 45, 90, or 180 mg $kg^{-1}$ b.w., [g]Not significant compared with FU (Tris) at 90 mg $kg^{-1}$ b.w., significant at $P<0.01$ compared with FU (Tris) at 180 mg $kg^{-1}$ b.w., [h]Not significant compared with FU (Tris) at 90 mg $kg^{-1}$ b.w., significant at $P<0.05$ compared with FU (Tris) at 180 mg $kg^{-1}$ b.w., [i]Not significant compared with FU (Tris) at 180 mg $kg^{-1}$ b.w., [j]Assuming that the concentration of Tris in a FU Roche vial is 84.7 mg $ml^{-1}$ (Matsubara *et al.*, 1980), this dose of Tris is equivalent to that brought by a FU (Tris) Roche vial in an experiment at 180 mg FU $kg^{-1}$ b.w., [k]Intentionally stopped at the mean time of ventricular arrest observed for a dose of 180 mg FU (Tris) $kg^{-1}$ b.w., [l]Not significant compared with control hearts, [m]This dose of FAC corresponds to the amount of Facet-Tris (oxazolidine 2) injected when hearts were treated with FU (Tris) Roche vials (batch B034S) at 180 mg FU $kg^{-1}$ b.w., The purity of FAC sodium salt was 95%, [n]Internationally stopped, [o]Since FBAL is very expensive and difficult to get, only one experiment was conducted with each dose, [p]This dose corresponds to the amount of Facet-Tris (oxazolidine 2) injected when hearts were treated with FU (Tris) Roche vials (batch B034S) at 180 mg FU $kg^{-1}$ b.w., [q]Significant at $P<0.0005$ compared with FU (Tris) at 180 mg $kg^{-1}$ b.w., [r]Significant at $P<0.005$ compared with FU (Tris) at 180 mg $kg^{-1}$ b.w., [z]Significant at $P<0.01$ compared with US FU (NaOH) Roche or Solopak, [t]Only two experiments were done with the German FU (NaOH) Roche since we could not get more vials of the same batch.

**Figure 2** $^{1}H$-decoupled $^{19}F$ NMR spectra of **a**, a perfusate from an isolated perfused rabbit heart treated with FU (Tris) Roche at 25 mg FU $kg^{-1}$ b.w. The pH was 7.93. The number of scans was 36775. **b**, a FU (Tris) Roche vial. The pH was 8.42. The number of scans was 368.

*What is the origin of FAC?*

We first surmised that it derived from the metabolic transformation of FBAL (Figure 1). However, there was no cardiotoxicity and no marked rise in intramyocardial citrate after treatment of IPRH with FBAL at 50 or 200 mg $kg^{-1}$ b.w. over 4 h (Table II). Only the signal of FBAL was found in the AS fractions of heart PCA extracts, demonstrating its effective uptake, and in the perfusates. No FAC was detected. These results show that, even in presence of high amounts of FBAL, this compound is not metabolised into FAC, at least not in amounts that can be detected by $^{19}F$ NMR.

We therefore concluded that FAC must be present in the injected solution, i.e. in FU Roche vials. The $^{19}F$ NMR analysis of FU (Tris) Roche vials of various lots revealed, besides FU itself, six fluorinated compounds accounting for $1.55 \pm 0.35$ mole% relative to FU ($n = 7$) (Table III, Figure 2b). Apart from the resonance at −43.2 ppm corresponding to fluoride ion, the other signals were unidentified. None of them corresponded to FAC (−141.0 ppm) or FC (−114.5 ppm, d, $J_{HF} = 50.7$ Hz).

FAC found in the perfusates of IPRH must therefore have come from transformation of impurity(ies) in the solution of FU. To check that the formation of FAC was a metabolic process rather than a chemical transformation taking place during the perfusion, control experiments consisting in perfusates containing FU from FU Roche vials and circulating during 2 or 4 h in the perfusion system without heart were carried out. The resonance of FU and those at −119.6, −121.3, −124.6, −126.0, −158.3 ppm were the only signals found in the $^{19}F$ NMR spectra of such perfusates. This also accounts for the unknown resonances observed in the spectra

**Table III** $^{19}F$ NMR chemical shifts, multiplicity and proportions of fluorinated impurities in FU (Tris) Roche vials of various batches[a] ($n = 7$)

| $\delta^{b,c}$ (ppm) | *Multiplicity*[d], *coupling constants J (Hz)* | *Proportion* ± *s.d. (mole %)* |
|---|---|---|
| −43.2 | s | 0.08±0.03 |
| −117.2[e] | dd, 48.5, 15.7 | –[f] |
| −122.3 | dd, 49.6, 18.4 | 0.52±0.08 |
| −124.8 | dd, 48.9, 22.5 | 0.05±0.01 |
| −125.9 | dd, 49.6, 20.6 | 0.57±0.16 |
| −159.7 | td, 46.8, 18.9 | 0.32±0.13 |

[a]Batches analysed: B034S, B035S, B044T, B045T, B048T, B049T, B051T, [b]Chemical shifts are related to external trifluoroacetic acid, [c]The pH of the vials was 8.45±0.16, [d]s = singlet, d = doublet, t = triplet, [e]Only detected in four out of seven vials analysed, [f]Too low to be accurately quantified.

of heart perfusates. FAC was not detected. FAC was therefore assumed to be formed via a metabolic process.

Various other experiments were conducted to verify that impurity(ies) was(were) the precursor of FAC and the causative factor of FU cardiotoxicity. No cardiotoxic symptoms were observed during the 4 h of perfusion of IPRH treated with FU Sigma at 25 or 180 mg $kg^{-1}$ b.w., a powder that we dissolved in Tris buffer and that contained no fluorinated impurities (Table II). Furthermore, there was no elevation in citrate levels with respect to the control ($P > 0.1$). For both doses, small signals of FUrd, FUPA, FBAL, $FUH_2$ were observed in the $^{19}F$ NMR spectra of the perfusates. The signal of FAC was undetected. At 180 mg FU $kg^{-1}$ b.w., we also observed a signal at −133.3 ppm corresponding to an unknown metabolite.

When vials of FU (Tris) Roche were treated in acidic conditions, FU precipitated and the $^{19}F$ NMR spectrum of the powder after its redissolution in Tris buffer showed no fluorinated impurity. IPRH were treated with this 'purified' solution at 180 mg FU $kg^{-1}$ b.w. No symptoms of cardiotoxicity appeared during the 4 h of perfusion. There was no significant intramyocardial accumulation of citrate ($P > 0.05$ *vs* control) and FAC was not detected in the perfusates (Table II).

The fact that the cardiotoxicity was due to impurity(ies) in FU Roche vials was confirmed by a last experiment with a sample of FU powder that Hoffmann-La Roche sent us and that we dissolved in Tris buffer immediately before the experiment. No fluorinated impurities were observed in the $^{19}F$ NMR spectrum of this solution. There was no evidence of cardiotoxicity, nor any rise in intramyocardial citrate (4 μg $g^{-1}$ of heart) and no detectable FAC in perfusates after treatment of IPRH with this solution at 180 mg FU $kg^{-1}$ b.w.

Therefore, FAC must have come from the metabolic transformation of degradation compound(s) contained in vials of FU Roche that is(are) formed during the storage of the solution.

### *Identification of the precursor of FAC in FU (Tris) Roche vials*

We noticed that the intensity of the signal at −159.7 ppm relative to those of the signals at −122.3 and −125.9 ppm was higher in the $^{19}F$ NMR spectra of FU (Tris) Roche vials than in those of perfusates of IPRH (compare Figures 2a and 2b). FAC was therefore assumed to be derived essentially from the metabolic conversion of the compound resonating at −159.7 ppm.

The simplest hypothesis was that FAC resulted from metabolic oxidation of Facet. We thus synthesised this compound by oxidation of fluoroethanol. In $H_2O$, it exists as the aldehyde hydrate 1. It reacts with Tris to give a compound (Facet-Tris) whose structure was determined as the oxazolidine 2, which on acid hydrolysis gives Facet hydrate 1 in the following reaction:

The compound resonating at −159.7 ppm was extracted from vials of FU (Tris) Roche. It had identical spectroscopic characteristics to those of the oxazolidine 2.

When IPRH were treated with this compound at an equivalent dose to that that would have been injected if hearts were treated with FU Roche vials at 180 mg $kg^{-1}$ b.w., ventricular arrest occurred at 140 ± 15 min, citrate levels rose significantly ($P < 0.0005$ *vs* control) and FAC was the main compound detected in the $^{19}F$ NMR spectra of perfusates in a similar amount to that found in perfusates of hearts treated with FU (Tris) Roche vials at 180 mg $kg^{-1}$ b.w. ($P > 0.1$) (Table II). The other small signals were unmetabolised oxazolidine 2 and Facet hydrate 1.

Therefore, a depot form of Facet that is converted into FAC by isolated perfused hearts is present in vials of FU (Tris) Roche.

### *$^{19}F$ NMR analysis of urine from patients treated with FU (Tris) Roche vials*

Thirty-three urine samples from 15 patients (six with cardiotoxic symptoms, nine without) treated with continuous i.v. infusion of FU (Tris) Roche were analysed. Unmetabolised FU and its classical catabolites ($FUH_2$, FUPA, FBAL and its derivatives, $F^-$) were observed in the $^{19}F$ NMR spectra. In addition, FAC was detected in all urine samples analysed irrespective of any reported cardiotoxicity. In some samples, it was also possible to detect the signals in the −120 ppm area found in FU vials. The signal of the oxazolidine 2 was not observed. A low signal of Facet hydrate was detected in some samples. A resonance with a $\delta$ varying between −153.0 and −154.5 ppm and attributed to a compound resulting from the chemical interaction of Facet with an unknown urinary component was observed in most samples. The quantitative analysis of urine samples from six patients showed that the ratio FAC excreted in 24 h/FU catabolites excreted in 24 h increased during the treatment for five patients (Figure 3).

**Figure 3** Ratios of FAC excreted over 24 h/FU catabolites excreted over 24 h in urine samples from six patients treated with a continuous i.v. infusion of FU (0.6 to 1 $g/m^2$/day) over 4 consecutive days. [a]No sample available.

Facet hydrate 1 Tris oxazolidine 2

FAC was not detected in the three plasma samples analysed. This could be explained by the relative insensitivity of NMR (detection threshold with our spectrometer 3–5 μM).

*FU (NaOH) vials*

To our knowledge, FU dissolved in Tris is used in France and Japan. In other countries (Germany, Great Britain, USA ...), FU vials contain FU dissolved in a sodium hydroxide solution. It was therefore interesting to analyse this kind of vials and to study their effect on IPRH. We analysed vials of FU manufactured by Roche in Great Britain, Germany or USA, as well as a US generic. All these vials gave similar $^{19}F$ NMR spectra (Figure 4b). The spectra were, however, more complex than those of the FU (Tris) vials (compare Figures 2b and 4b). $F^-$ was the main impurity (representing≈0.3–1.5 mole% relative to FU) but signals from approximately 50 different fluorinated substances could be observed at individual concentrations of approximately 0.005–0.1 mole%. The total of the combined fluorinated impurities came to between 1 and 3 mole%. Since these vials do not contain Tris, any Facet present should be in the form of the hydrate 1 and not the oxazolidine 2. We therefore sought Facet hydrate. There was a marked increase in the signal at −155.16 ppm after spiking the sample with Facet hydrate, although more detailed examination of this signal (which represents ≈0.01–0.05 mole%) showed two close signals (Δυ≈3 Hz), one of which was Facet hydrate (Figure 4b insert). This impurity could not therefore be quantified accurately.

IPRH were treated with the content of these vials at 180 mg FU $kg^{-1}$ b.w. Ventricular arrest occurred at various times even in experiments with vials of the same origin (Table II). However, we consistently observed episodes of arrythmia after around 2 h. The German FU led to a marked rise in intramyocardial citrate, comparable to that observed with FU (Tris) Roche. There was a lower but significant rise in citrate levels after treatment with the contents of the American and English FU vials ($P<0.0005$ *vs* control). FAC

**Figure 4** $^1H$-decoupled $^{19}F$ NMR spectra of **a**, a perfusate from an isolated perfused rabbit heart treated with FU (NaOH) Roche manufactured in Germany at a dose of 180 mg FU $kg^{-1}$ b.w. The pH was 7.38. The number of scans was 46313. **b**, a vial of FU (NaOH) Roche manufactured in Germany. The pH was 8.5. The number of scans was 35324.

was detected in all perfusates analysed irrespective of the origin of the injected vial (Figure 4a), but in a significantly lower amount than that observed in perfusates of hearts treated with FU (Tris) at the same dose ($P<0.005$ for American and English FU vials *vs* FU (Tris) vials). However, the amount of FAC was higher in the perfusates of hearts treated with German and English FU than in those of hearts treated with US FU ($P<0.01$ for English FU *vs* US FU).

### Discussion

The original aim of this study was to relate the cardiotoxicity of FU to metabolism of the drug in the heart. Indeed, Japanese workers had reported that the ECG changes elicited by i.v. administration of FU to the guinea pig were associated with an intracellular depletion of high-energy phosphate compounds and a substantial elevation of intramyocardial citrate level (Matsubara *et al.*, 1980). They suggested that the accumulation of citrate was due to the formation of FAC from FU via FBAL. Formation of FAC from FBAL has also been proposed as a biochemical basis for FU-related neurotoxicity (Philips *et al.*, 1959; Koenig & Patel, 1970). However, attempts to demonstrate the presence of FAC in acid-soluble extracts of mouse liver and kidney and in the urine of mice, cats or humans given FU have so far been unsuccesful (Mukherjee & Heidelberger, 1960; Hull *et al.*, 1988). Also, no metabolism of [2-$^{14}$C]FBAL into FAC has been observed in mice and cats (Mukherjee & Heidelberger, 1960). So, the detection of FAC in perfusates of isolated rabbit hearts treated with FU vials (Figures 2a and 4a) was surprising, especially as significant amounts of FAC, but not of the usual metabolites of FU, were detected in the experiments with FU (Tris) Roche at 180 mg kg$^{-1}$ b.w. Moreover, since FBAL was not converted into FAC by isolated heart, the significant amounts of FAC observed in our experiments could not have come from metabolism of FU via FBAL.

The lack of symptoms of cardiotoxicity and the absence of FAC in perfusates of hearts treated with FU powder (Sigma, Roche) dissolved just before use suggested that the origin of FAC had to be searched in the injected vials. FU vials contain fluorinated impurities whether NaOH or Tris solutions are used to dissolve the drug (Figures 2b and 4b). Fluoride ion was found in both types of vial. A critical impurity was identified in the FU Tris vials, namely the oxazolidine of Facet. It is known that Tris reacts readily with propionaldehyde or aldoifosfamide to produce the corresponding oxazolidines (Borch & Getman, 1984; Zon *et al.*, 1984). We demonstrated by synthesis and isolation of this impurity and with conventional spectroscopic techniques that Tris also reacts with Facet to give the fluorinated oxazolidine 2. Injected into IPRH, it is metabolised into Facet and FAC. Thus, a depot form of Facet is present in FU (Tris) vials. It was noteworthy, however, that the ventricular arrest induced by the FU (Tris) Roche vials injected into IPRH at a dose of 180 mg FU kg$^{-1}$ b.w. occurred significantly earlier ($P<0.0005$) than that induced by the oxazolidine 2 injected alone at a dose equivalent to that injected with FU Tris vials for a dose of 180 mg FU kg$^{-1}$ b.w. (70 and 140 min respectively). Since a freshly made up solution of FU did not induce symptoms of cardiotoxicity under our experimental conditions, it is likely that the impurities giving signals at $-122.3$ and $-125.9$ ppm have some responsibility for the toxicity although probably not via FAC since the amount of FAC detected did not differ significantly between hearts treated with FU (Tris) vials at 180 mg kg$^{-1}$ b.w. and those treated with oxazolidine 2 at a dose equivalent to that injected with FU Tris vials for a dose of 180 mg FU kg$^{-1}$ b.w. In FU NaOH vials, we could identify Facet, and many unidentified fluorinated impurities were also detected.

These impurities are degradation products of FU, which form during storage in basic medium. To our knowledge, there are no detailed reports of the stability of FU or of identification of its products of decomposition. However, it has been reported that FU degrades more rapidly in alkaline than in weakly acidic conditions (Garrett *et al.*, 1968; Tomankova & Zyka, 1977). In preliminary experiments, we examined the stability of FU at a concentration of 50 mg ml$^{-1}$ in Tris solution (1 M) at pH 8.5 and 25°C (data not shown). The signals at $-122.3$ and $-125.9$ ppm were detected within 4 days after dissolving FU powder in Tris; by day 14, we also observed the signals of fluoride ion and the compounds resonating at $-117.2$ and $-124.8$ ppm, and by day 20, the signal of the oxazolidine 2. A more detailed study of the mechanism of the alkaline hydrolysis of FU is in progress. The decomposition of FU in alkaline medium which is required for solubilisation leads to a compound that is metabolised to highly toxic FAC (LD 50 = 0.1–7.5 mg kg$^{-1}$ b.w. depending on the species (Meldrum & Bignell, 1957)). FAC exerts specific actions on the myocardium and nervous system causing ventricular fibrillation and/or convulsions (Chenoweth & Gilman, 1946). To avoid any possibility of chemical degradation, the solution of FU should be prepared immediately before injection. Modification of the manufacturing procedure for FU vials (a lyophilised form for example) should help limit the cardiotoxicity (and maybe also the neurotoxicity) of this drug.

FAC was consistently detected in the urine of patients treated with high doses of FU Tris Roche, the only formulation and brand available in France. There appeared to be no relationship, however, between urinary levels and clinical cardiotoxic symptoms. Various factors could account for this. Variations in the amounts of FAC, either from differences in amounts of Facet injected depending on the injected batch, or from individual differences in metabolising Facet or in defluorinating FAC, the main metabolic pathway of FAC detoxification (Mead *et al.*, 1979; Tecle & Casida, 1989), the existence of underlying myocardial disease could account for the incidence of ≈10% reported for FU-related cardiotoxicity in most clinical studies. We noticed an increase in the daily urinary excretion of FAC during perfusion of FU in five out of the six patients studied (Figure 3). This could account for one of the striking features of FU cardiotoxicity namely the delayed onset with respect to the beginning of the treatment of clinical cardiotoxic symptoms (Collins & Weiden, 1987; Freeman & Costanza, 1988; de Forni *et al.*, 1990; 1991).

Although our results are preliminary, we did observe differences in cardiotoxic effects and citrate levels between the German, English and American FU NaOH vials (Table II). Since the $^{19}$F NMR spectra from these formulations are similar but very complex, we cannot readily account for these diverse data. Work is in progress to elucidate the matter.

We would like to thank Dr R. Lehr for his helpful assistance.

This study was supported by grants from the Association pour la Recherche sur le Cancer (ARC, Villejuif, France) and Ligue Nationale Française contre le cancer (Comité des Hautes-Pyrénées, Tarbes, France).

### References

BORCH, R.F. & GETMAN, K.M. (1984). Base-catalyzed hydrolysis of 4-hydroperoxycyclophosphamide: evidence for iminocyclophosphamide as an intermediate. *J. Med. Chem.*, **27**, 485–490.

CHENOWETH, M.B. & GILMAN, A. (1946). Studies on the pharmacology of fluoroacetate. I. Species response to fluoroacetate. *J. Pharmacol. Exper. Ther.*, **87**, 90–103.

COLLINS, C. & WEIDEN, P.L. (1987). Cardiotoxicity of 5-fluorouracil. *Cancer Treat. Rep.*, **71**, 733–736.

DE FORNI, M., BUGAT, R., SORBETTE, F., DELAY, M., BACHAUD, J.M. & CHEVREAU, C. (1990). Cardiotoxicité du 5-fluorouracile perfusion intraveineuse continue: étude clinique, prévention, physiopathologie. A propos d'une série de 13 cas. *Bull. Cancer*, **77**, 429–438.

DE FORNI, M., JAILLAIS, P., LEMAIRE, L., BACHAUD, J.M., BOUDJEMA, B., CHEVREAU, C., MALET-MARTINO, M.C, DUDOUET, P., DAVID, J.M., ROCHE, H., MARTINO, R. & BUGAT, R. (1991). Cardiotoxicity of high-dose 5-fluorouracil: a prospective clinical study with biochemical approach using fluorine-19 nuclear magnetic resonance. *Proc. ECCO.*, **6**, 1980.

DENT, R.G. & MCCOLL, I. (1975). 5-fluorouracil and angina. *Lancet*, 347–348.

ENSLEY, J.F., PATEL, B., KLONER, R., KISH, J.A., WYNNE, J. & AL-SARRAF, M. (1989). The clinical syndrome of 5-fluorouracil cardiotoxicity. *Investig. New Drugs*, **7**, 101–109.

ESKILSSON, J., ALBERTSSON, M. & MERCKE, C. (1988). Adverse cardiac effects during induction chemotherapy treatment with cisplatin and 5-fluorouracil. *Radiother. Oncol.*, **13**, 41–46.

FREEMAN, N.J. & COSTANZA, M.E. (1988). 5-fluorouracil-associated cardiotoxicity. *Cancer*, **61**, 36–45.

GARRETT, E.R., NESTLER, H.J. & SOMODI, A. (1968). Kinetics and mechanisms of hydrolysis of 5-halouracils. *J. Org. Chem.*, **33**, 3460–3468.

GRADISHAR, W.J. & VOKES, E.E. (1990). 5-fluorouracil cardiotoxicity: a critical review. *Annals Oncol.*, **1**, 409–414.

GRADISHAR, W.J., VOKES, E.E., SCHILSKY, R.L., WEICHSELBAUM, R. & PANJE, W. (1990). Catastrophic vascular events in patients receiving 5-fluorouracil (5-FU) based chemotherapy. *Proc. Amer. Assoc. Cancer Res.*, **31**, 1128.

HEIDELBERGER, C., DANENBERG, P.V. & MORAN, R.G. (1983). Fluorinated pyrimidines and their nucleosides. *Adv. Enzymol. Relat. Areas. Mol. Biol.*, **54**, 57–119.

HULL, W.E., PORT, R.E., HERRMANN, R., BRITSCH, B. & KUNZ, W. (1988). Metabolites of 5-fluorouracil in plasma and urine, as monitored by $^{19}F$ nuclear magnetic resonance spectroscopy, for patients receiving chemotherapy with or without methotrexate pretreatment. *Cancer Res.*, **48**, 1680–1688.

KOENIG, H. & PATEL, A. (1970). Biochemical basis for fluorouracil neurotoxicity. *Arch. Neurol.*, **23**, 155–160.

LABIANCA, R., BERETTA, G., CLERICI, M., FRASCHINI, P. & LUPORINI, G. (1982). Cardiac toxicity of 5-fluorouracil: a study on 1083 patients. *Tumori*, **68**, 505–510.

MALET-MARTINO, M.C., ARMAND, J.P., LOPEZ, A., BERNADOU, J., BETEILLE, J.P., BON, M. & MARTINO, R. (1986). Evidence for the importance of 5′-deoxy-5-fluorouridine catabolism in humans from $^{19}F$ nuclear magnetic resonance spectrometry. *Cancer Res.*, **46**, 2105–2112.

MALET-MARTINO, M.C, MARTINO, R. & ARMAND, J.P. (1990). La spectroscopie de résonance magnétique nucléaire du fluor-19: un outil privilégié pour l'étude du métabolisme et de la pharmacocinétique des fluoropyrimidines. *Bull Cancer*, **77**, 1223–1244.

MALET-MARTINO, MC. & MARTINO, R. (1991). Uses and limitations of nuclear magnetic resonance spectroscopy in clinical pharmacokinetics. *Clin. Pharmacokin.*, **20**, 337–349.

MARTINO, R., MALET-MARTINO, M.C., VIALANEIX, C., LOPEZ, A. & BON, M. (1987). $^{19}F$ NMR analysis of the carbamate reaction of α-fluoro-β-alanine, the major catabolite of fluoropyrimidines. Application to FBAL carbamate determination in body fluids of patients treated with 5′-deoxy-5-fluorouridine. *Drug Metab. Dispos.*, **15**, 897–904.

MATSUBARA, I., KAMIYA, J. & IMAI, S. (1980). Cardiotoxic effects of 5-fluorouracil in the guinea pig. *Japan J. Pharmacol.*, **30**, 871–879.

MEAD, R.J., OLIVER, A.J. & KING, D.R. (1979). Metabolism and defluorination of fluoroacetate in the brush-tailed possum (*Trichosurus vulpecula*). *Aust. J. Biol. Sci.*, **32**, 15–26.

MELDRUM, G.K. & BIGNELL, J.T. (1957). The use of sodium fluoracetate (compound 1080) for the control of the rabbit in Tasmania. *Aust. Veter. J.*, **33**, 186–196.

MUKHERJEE, K.L. & HEIDELBERGER, C. (1960). Studies on fluorinated pyrimidines. IX. The degradation of 5-fluorouracil-6-$C^{14}$. *J. Biol. Chem.*, **235**, 433–437.

PARISOT, D., MALET-MARTINO, M.C., MARTINO, R. & CRASNIER, P. (1991). $^{19}F$ nuclear magnetic resonance analysis of 5-fluorouracil metabolism in four differently pigmented strains of *Nectria haematococca. Applied Environm. Microbiol.*, **57**, 3605–3612.

PATTISON, F.L.M. & PETERS, R.A. (1966). Monofluoro aliphatic compounds. In *Handbook of Experimental Pharmacology*, Smith, F.A. (ed.) p. 387–458. Springer-Verlag: New York.

PHILIPS, F.S., DUSCHINSKY, R. & STERNBERG, S.S. (1959). Pharmacology of 5-fluorinated pyrimidines. *Proc. Amer. Assoc. Cancer Res.*, **3**, 51.

REZKALLA, S., KLONER, R.A., ENSLEY, J., AL-SARRAF, M., REVELS, S., OLIVENSTEIN, A., BHASIN, S., KERPEL-FRONIOUS, S. & TURI, Z.G. (1989). Continuous ambulatory ECG monitoring during fluorouracil therapy: a prospective study. *J. Clin. Oncol.*, **7**, 509–514.

SUZUKI, T., NAKANISHI, H., HAYASHI, A., NAKAHATA, N., TAKANO, S. & ITO, G. (1977). Cardiac toxicity of 5-fluorouracil in rabbits. *Japan J. Pharmacol.*, **27**, suppl. 137P.

TECLE, B. & CASIDA, J.E. (1989). Enzymatic defluorination and metabolism of fluoroacetate, fluoroacetamide, fluoroethanol, and (−)-*erythro*-fluorocitrate in rats and mice examined by $^{19}F$ and $^{13}C$ NMR. *Chem. Res. Toxicol.*, **2**, 429–435.

TOMANKOVA, H. & ZYKA, J. (1977). Study of the stability of pyrimidine series cytostatics, ftorafur and fluorouracil injections. *Microchem. J.*, **22**, 70–84.

WAIN, W.H. & STAATZ, W.D. (1973). Rates of synthesis of ribosomal protein and total ribonucleic acid through the cell cycle of the fission yeast Schizosaccharomyces pombe. *Exp. Cell Res.*, **81**, 269–278.

ZON, G., LUDEMAN, S.M., BRANDT, J.A., BOYD, V.L., OZKAN, G., EGAN, W. & SHAO, K. (1984). NMR spectroscopic studies of intermediary metabolites of cyclophosphamide. A comprehensive kinetic analysis of the interconversion of *cis*- and *trans*-4-hydroxycyclophosphamide with aldophosphamide and the concomitant partitioning of aldophosphamide between irreversible fragmentation and reversible conjugation pathways. *J. Med. Chem.*, **27**, 466–485.